# Complete Genome Sequence of a *bla*_CTX-M-1_-Harboring *Escherichia coli* Isolate Recovered from Cattle in Germany

**DOI:** 10.1128/genomeA.01476-17

**Published:** 2018-01-25

**Authors:** Jens A. Hammerl, Alexandra Irrgang, Mirjam Grobbel, Bernd-Alois Tenhagen, Annemarie Käsbohrer

**Affiliations:** a German Federal Institute for Risk Assessment (Bundesinstitut für Risikobewertung [BfR]), Department Biological Safety, Unit Epidemiology, Zoonoses and Antimicrobial Resistance, Berlin, Germany; b Institute for Veterinary Public Health, University of Veterinary Medicine, Vienna, Austria

## Abstract

We describe here the whole-genome sequence and basic characteristics of *Escherichia coli* isolate 15-AB01393, recovered from German beef within a national monitoring program in 2015. This isolate was identified as an extended-spectrum-β-lactamase-producing *E. coli* strain of multilocus sequence type (MLST) ST58 harboring the antimicrobial resistance genes *bla*_CTX-M-1_, *mph*(A), *sul2*, *dfrA5*, *strA*, and *strB*.

## GENOME ANNOUNCEMENT

Extended-spectrum β-lactamases (ESBL) confer resistance to third-generation cephalosporins. ESBL-producing bacteria are a major public health issue, as third-generation cephalosporins are considered by the WHO to be highest-priority critically important antimicrobials (1). These bacteria can be transmitted from animals to humans via contaminated food products or contact with food-producing animals (2). Dissemination of ESBL-producing bacteria mainly occurs by the transfer of plasmids of different incompatibility groups (3) but also vertically via clonal spread (4, 5). In contrast to humans, where CTX-M-15 is the dominant ESBL type, CTX-M-1 is most common type in livestock (6, 7).

To support efficient risk management strategies for the control of ESBL-producing bacteria in food-producing animals and food, the genetic basis of selected commensal *Escherichia coli* strains from the German national monitoring program for antimicrobial resistance in zoonotic agents in the food chain was investigated by the National Reference Laboratory for Antimicrobial Resistance (NRL-AR).

In this study, the genome sequence of the *E. coli* isolate 15-AB01393, recovered from German beef, was determined. This isolate exhibits a non-wild-type phenotype for ampicillin (MIC, >64 mg/liter), azithromycin (MIC, 64 mg/liter), sulfamethoxazole (MIC, >1,024 mg/liter), cefepime (MIC, 32 mg/liter), ceftazidime (MIC, 2 mg/liter), and cefotaxime (MIC, 64 mg/liter) using the microdilution method, according to CLSI guidelines (8), using EUCAST epidemiological cutoff values (http://www.eucast.org/clinical_breakpoints/). A single colony from MacConkey agar with 1 mg/liter cefotaxime was cultured in lysogeny broth for 24 h at 37°C. Genomic DNA was isolated from the liquid culture using the PureLink genomic DNA minikit (Invitrogen, Carlsbad, CA, USA) and used for the generation of a Nextera XT library (Illumina, CA, USA). Genome sequencing of 2 × 250-bp paired-end reads was conducted on an in-house Illumina MiSeq sequencing platform (9). A *de novo* genome assembly (total genome length, 5,055,005 bp; *N*_50_ contig length, 59,668 bp) was performed using SPAdes (version 3.5.0) of the PATRIC database (10), resulting in 279 contigs with >25-fold sequence coverage per consensus base. Genome analysis using Web-based tools of the Center for Genomic Epidemiology (https://cge.cbs.dtu.dk/services/) revealed that the *E. coli* isolate belongs to the multilocus sequence type (MLST; Achtman scheme) ST58 (clonal complex 155 [CC155]) (MLST 1.8) (11) and harbors genes (*wzt*, *wzm*, and *fliC*) specific for the O8:H25 serotype (SerotypeFinder 1.1) (12) and the FimH-type determinant *fimH32* (FimTyper 1.0; https://cge.cbs.dtu.dk/services/FimTyper-1.0/). Furthermore, ResFinder 3.0 analysis (13, 14) revealed the presence of the resistance genes *bla*_CTX-M-1_ (β-lactam), *mph*(A) (macrolide, lincosamide, and streptogramin B), *sul2* (sulfonamide), *dfrA5* (trimethoprim), and the aminoglycoside determinants *strA* and *strB*. Additionally, several nucleotide variations in the *ampC*, *gyrB*, *parC*, *pmrB*, 16S *rrsB*, 16S *rrsC*, 16S *rrsH*, and 23S genes were detected, which may also contribute to the observed antimicrobial resistance phenotype. Initial genome annotation using the NCBI Prokaryotic Genome Annotation Pipeline (released 2013) (15) resulted in the detection of 5,453 genes, 19 rRNAs (5S, 16S, and 23S), 83 tRNAs, 9 noncoding RNAs (ncRNAs), 167 pseudogenes, and 2 clustered regularly interspaced short palindromic repeat (CRISPR) arrays. Bioinformatically, four contigs were identified harboring conserved target sequences of the plasmid-based incompatibility groups IncX1 (98.66%), IncQ1 (100%), IncFII (100%), and IncFIB (AP001918) (98.39%) (PlasmidFinder 1.3) (16). Further examinations are planned to determine the impact of the isolate for the distribution of the resistance genes.

### Accession number(s).

The whole-genome sequence of *E. coli* isolate 15-AB01393 was deposited in GenBank under the accession number PEAV00000000.
